# Losing is the new winning: loss of histone demethylase DT2 enhances drought stress tolerance in rice.

**DOI:** 10.1093/plphys/kiaf328

**Published:** 2025-07-23

**Authors:** Gunjan Sharma

**Affiliations:** Assistant Features Editor, Plant Physiology, American Society of Plant Biologists; School of Biosciences, University of Birmingham, Edgbaston B15 2TT, UK

Rice is a staple cereal crop for more than half of the world's population. Rice, a semi-aquatic crop, needs continuous irrigation. Climate change has increased the frequency of droughts during recent years leading to reduced yield. Plants have evolved intricate, molecular signaling cascades involving molecular components to combat drought stress. Regulation of abscisic acid (ABA) biosynthesis and signalling is a conserved component for physiological changes conferring drought stress tolerance (Zhu 2002; Tardieu et al. 2018).

DNA packaging in the nucleus regulates chromatin accessibility affecting transcription. During stress, nucleosomes dynamically change chromatin accessibility through addition of transcriptionally active (H4R3me2, H3K4me3 and H3K36me2/3) and repressive (H4R3me2, H3K9me2/3 and H3K27me3) marks on histone tails (Liu et al. 2022). Reduced chromatin accessibility leads to reduced gene expression. Reversible addition of histonemarks is regulated by writers and erasers which can be methyltransferases and demethylases, respectively. Studies in the model plant *Arabidopsis* have demonstrated the fine tuning of developmental transitions and stress responses through epigenetic plasticity (Yu et al. 2025). The full potential of histone modifiers in enhancing abiotic stress tolerance in crop species such as rice is not fully understood.

In a recently published article in *Plant Physiology*, Wang et al. (2025) have identified a novel histone demethylase DROUGHT TOLERENCE 2 (DT) acting as a negative regulator of drought stress tolerance by dampening ABA biosynthesis. The authors revealed that DT2 reduces ABA content by activating the expression of *OsZIP26*, a negative regulator of ABA biosynthesis through a targeted removal of H3K9me2 repressive marks, and simultaneously interacting with an ABA stress-ripening–inducible 5 protein (ASR5), thereby restricting ASR5-mediated ABA biosynthesis.

To identify the potential candidate gene for drought tolerance, the authors screened rice mutant lines generated by gamma ray exposure (Sun et al. 2024). Gamma ray induced heritable changes in DNA is a widely employed breeding strategy for trait improvement incrops (Oladosu et al. 2016). One of the mutant lines M28 exhibited a strong drought tolerance phenotype. To identify the gene responsible, a map-based cloning approach narrowed the search to three genes. Sequencing of these genes identified a mutation (a 28-base pair deletion in 9th exon) in a previously unknown gene *DT2*. The authors confirmed the role of *DT2* in drought response by showing that *DT2* CRISPR-knockout mutants conferred drought tolerance, while overexpression led to sensitivity.

To further elucidate the molecular function of *DT2* gene, Wang et al. (2025) studied its expression profiles in a tissue-specific and drought stress–responsive manner. The *DT2* was expressed in all rice tissues with highest expression in panicles. Interestingly, *DT2* expression was induced initially upon water deprivation and subsequently reduced after a prolonged drought stress exposure suggesting a possible negative feedback loop. The DT2 protein exhibited nuclear localization and contained an N-terminal RING finger and a C-terminal JmjC domain. The demethylase activity of the JmjC domain, was confirmed by quantifying H3K9me2 methylation marks in WT and DT2 mutants. As expected, DT2 knockout resulted in an overall increase in H3K9me2 methylation. Intriguingly, *OsZIP26* was revealed as one of the DT2 target genes; OsbZIP26 was previously reported to be anegative regulator of ABA signalling (Duan et al. 2023). Increased H3K9me2 methylation leading to reduced expression of *OsZIP26* was confirmed in *DT2* knockout mutants through chromatin immunoprecipitation (ChIP)-qPCR. In addition, *OsZIP26* CRISPR-knockout mutants showed improved drought tolerance. These compelling observations suggested that *DT2*-mediated positive regulation of *OsZIP26* reduces ABA signallingunder well-watered conditions.

To further reveal the likely downstream targets of OsZIP26 transcription factor, the authors compared transcriptomes of WT and *DT2* mutant rice under drought stress. An interesting candidate gene, *bHLH048* exhibited reduced expression in *DT2* mutant lines.The direct targeting of *bHLH048* by *OsZIP26* was confirmed by yeast one-hybrid and ChIP-qPCR. The dual-luciferase assay showed that *OsZIP26* induces *bHLH048* expression. Interestingly, *bHLH048* reduces the expression of the ABA biosynthesis gene *NCED2* through direct physical interaction with the promoter region (Xu et al. 2022). The authors showed increased expression of *NCED2* gene in *OsZIP26* CRISPR mutants. It was speculated that *OsZIP26* might reduce ABA levels by reducing *NCED2* expression through activation of repressive transcription factor bHLH048. DT2-mediated regulation of drought stress tolerance through a module of OsZIP26, bHLH048 and yet unknown players warrant experiments focusing on additional DT2 demethylation targets.

To hunt for novel components of DT2-mediated regulation of drought stress tolerance, Wang et al. (2025) revealed the potential interactors of DT2 through the yeast two-hybrid system. Screening revealed an attractive candidate protein, transcription factor ASR5. The interaction of the RING domain of DT2 with ASR5 occurred in the nucleus. Phenotypic analysis showed that ASR5 knockout mutants were sensitive to drought stress suggesting ASR5 acts as a positive regulator of drought stress tolerance response. Rice ASR5 enhances drought tolerance through a stomatal closure pathway associated with ABA signalling (Li et al. 2017). The authors further explored the role of ASR5 in ABA signalling and found that ASR5 binds to the *NCED2* promoter ultimately affecting the ABA content. Interestingly, DT2 hampers the binding of ASR5 to the *NCED* promoter through a yet unknown mechanism.

In conclusion, Wang et al. 2025 have delineated the role of a novel histone demethylase DT2 in fine-tuning the rice drought stress tolerance response via a signalling cascade consisting of molecular regulators OsZIP26, bHLH048, and ASR5 (Figure.). Since the expression of *DT2* is reduced during prolonged drought stress, it would be interesting to investigate the post-translational regulation of DT2. Furthermore, investigations on the possibility of DT2-derived drought stress transgenerational memory via known target OsZIP26 and other novel targets would be fascinating. Stress tolerance is usually associated with yield penalty. Promisingly, DT2 mutants did not compromise yield, making it an attractive potential candidate gene for improving drought tolerance through breeding strategies in rice.

**Figure.**
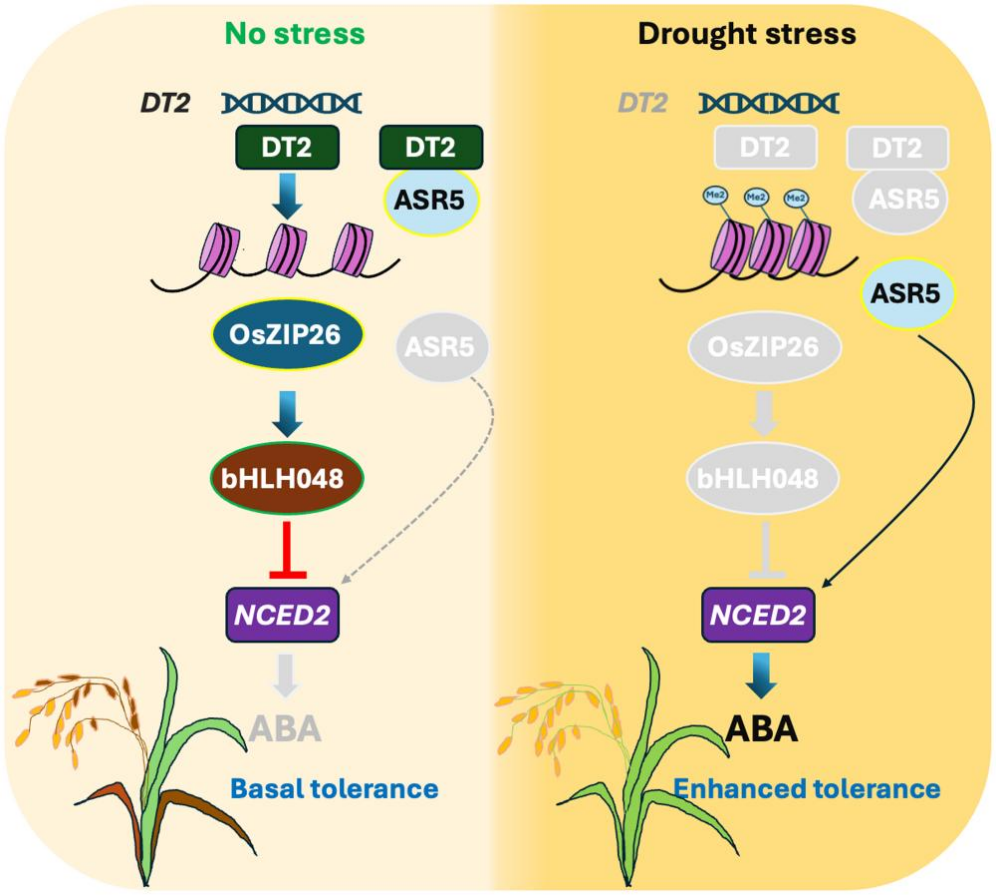
A Histone demethylase DT2 fine tunes the rice drought stress tolerance response via a signalling cascade comprised of molecular regulators OsZIP26, bHLH048 and ASR5. The DT2 protein contain an N-terminal RING finger and a C-terminal JmjC domain. During unstressed conditions, the JmjC domain-mediated DT2 demethylase activity limits excessive ABA accumulation by removing H3K9me2 histone methylation on *OsZIP26*. An increased *OsZIP26* expression induces *bHLH048* reducing *NCED2* expression and ABA biosynthesis. Additionally, RING finger domain of DT2 interacts with ASR5 thereby reducing ASR5-mediated induction of *NCED2*. However, during prolonged drought stress, reduced *DT2* expression allows H3K9me2 to persist on *OsZIP26* reducing *bHLH048* induction thereby easing the repression of ABA biosynthesisand enabling ASR5-mediated *NCED2* induction. An increased *NCED2* expression in turn increases ABA accumulation ultimately providing improved drought stress tolerance (adapted from Wang et al. 2025). Grey scale components depict reduction in activity.

## Data Availability

No data were generated or analyzed in this study.
